# Patterning of mutually interacting bacterial bodies: close contacts and airborne signals

**DOI:** 10.1186/1471-2180-10-139

**Published:** 2010-05-12

**Authors:** Jaroslav J Čepl, Irena Pátková, Anna Blahůšková, Fatima Cvrčková, Anton Markoš

**Affiliations:** 1 Department of philosophy and history of science, Charles University in Prague, Faculty of Science, Viničná 7, Praha 2, 128 44 Czechia; 2 Department of experimental plant biology, Charles University in Prague, Faculty of Science, Viničná 5, Praha 2, 128 44 Czechia

## Abstract

**Background:**

Bacterial bodies (colonies) can develop complex patterns of color and structure. These patterns may arise as a result of both colony-autonomous developmental and regulatory processes (self-patterning) and environmental influences, including those generated by neighbor bodies. We have studied the interplay of intra-colony signaling (self-patterning) and inter-colony influences in related clones of *Serratia rubidaea *grown on rich media.

**Results:**

Colonies are shaped by both autonomous patterning and by signals generated by co-habitants of the morphogenetic space, mediating both internal shaping of the body, and communication between bodies sharing the same living space. The result of development is affected by the overall distribution of neighbors in the dish. The neighbors' presence is communicated via at least two putative signals, while additional signals may be involved in generating some unusual patterns observed upon encounters of different clones. A formal model accounting for some aspects of colony morphogenesis and inter-colony interactions is proposed.

**Conclusions:**

The complex patterns of color and texture observed in *Serratia rubidaea *colonies may be based on at least two signals produced by cells, one of them diffusing through the substrate (agar) and the other carried by a volatile compound and absorbed into the substrate. Differences between clones with regard to the interpretation of signals may result from different sensitivity to signal threshold(s).

## Background

Bacteria can display a plethora of multicellular forms (colonies, mats, stromatolites, etc.); their structure and appearance depends on factors such as the presence of nutrients or neighbors. Concepts of "body" and "community", as developed for multicellular sexual eukaryots, became, however, somewhat blurred upon attempts of their application to microorganisms. Is differentiation of multicellular units in bacteria comparable to embryonic development, to the establishment of an ecosystem? Is it even the place of Darwinian evolution on a micro-scale?

Multicellular bacterial bodies can be viewed as ecosystems negotiated by myriads of (presumably genetically different and selfish) specialists (e.g. [1-6]). Each cell is understood as an individual playing its own game according to resources, energy costs, and complicated informational interactions with others. However, patterning of multicellular bodies remains beyond interest, at the most being viewed as a passive outcome of physical forces.

A multicellular bacterial community may, however, be also perceived as the prevalent form of bacterial existence, with a genuine ontogeny. To create and maintain such elaborated structures, a great deal of communication, regulations, mutual understanding, and cooperation takes place in bacterial morphogenesis. Differentiation in such a bacterial body (*as *a body, not a population of cells) may proceed via genetically differing subclones fulfilling different roles, and appearing reproducibly at characteristic periods of cultivation [7-10]. Sophisticated networks of chemical signals [11-13], the scaffolding of extracellular matrix [14] and even cell-to-cell contacts [15,16] may enable attaining and maintaining the integrity of the body. Research in this direction has been greatly accelerated in last two decades by the discovery of the phenomenon of quorum sensing (see [17-19]; for *Serratia *see [13]). Bacterial populations react to such signals - and build multispecies bodies accordingly - in a context-dependent manner [20]. A plethora of quorum-modulating signals, such as indole or furanole derivatives, was also described [12,21,22]. The study of model monoclonal populations may contribute to understanding colony morphogenesis, providing the possibility to examine how, and even why, bacteria exert themselves towards "species-specific" appearances.

We have previously demonstrated that colonies of *Serratia marcescens *can be viewed as multicellular bodies with genuine embryonic development [23]. Colonies displayed finite growth and clone-specific formative processes; even a disorganized cell slurry (up to 10^7 ^cells) could establish a regular pattern and embark on a typical developmental pathway. Under standardized culture conditions on rich semi-solid media, the final shape and patterning of bacterial bodies depended essentially on four initial settings: (1) amount, density, and distribution pattern of founder cells; (2) the configuration of surrounding free medium; (3) the presence and character of other bacterial bodies sharing the same niche; and (4) self-perception, resulting in delimitation towards other bodies.

Here we further investigate the development of bacterial bodies and their interaction with close or distant neighbors of identical, or different, clonal origin. We also propose a formal model that can account for some of our experimental results.

## Results

### Colony patterning in clonal variants of *Serratia rubidaea*

**Figure 1:**
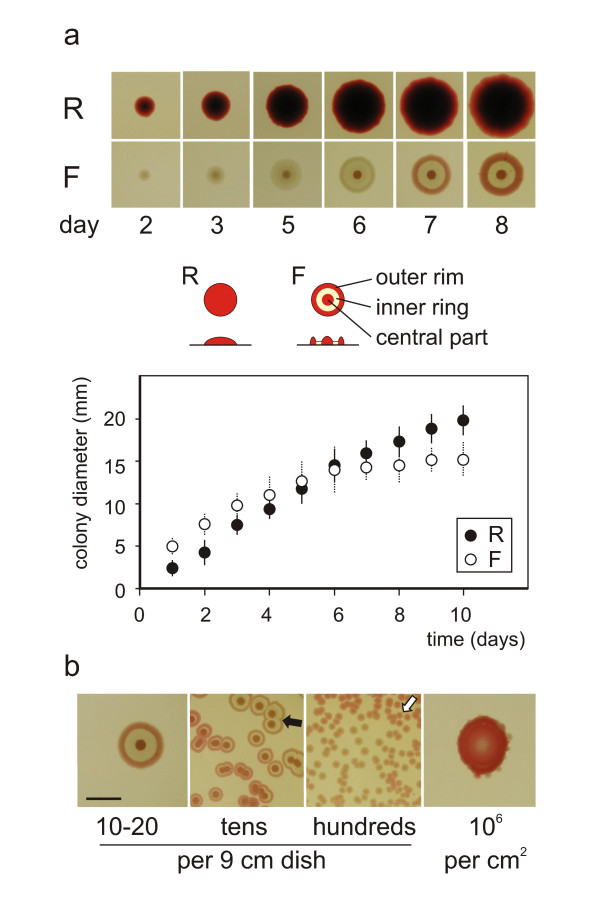
**Summary of clone phenotypes under various growth conditions**. **a**. Comparison of two basic phenotypes: **R **(rimless "wild type") and **F **(rimmed) Top: appearance of colonies at given time-points; middle - sketches (contours and cross-sections) of fully developed colonies; bottom - time-course of colony growth (N = 10-16 for each point, +/- SD). **b**. Dependence of colony patterning (7 days old) on the density of planting (shown below the figures; bar = 1 cm). Note confluent colonies characteristic by their separate centers and common rim (black arrow), undeveloped (dormant) forms (white arrow), and an undifferentiated macula formed at high plating density (right).

We have chosen a wild type strain of *Serratia rubidaea*, a Gram-negative, facultatively anaerobic, rod-shaped bacterium of the Enterobacteriaceae family ([24]; see Methods), producing usually red glossy colonies without any distinguished structural pattern except of a slightly darker touch in the middle, as our starting material. This strain will be further referred to as the **R **(Red) strain. The **R **strain occassionally segregated colonies of variant shape or colouring upon prolonged cultivation in liquid media and serial plating; from these, we selected three stable, no longer segregating clones used in this study: (1) **W **(White), a colorless variant of **R**; (2) **F **(Fountain) clone named after its cross-section profile (Figure 1a); (3) **Fw **(Fountain white) - a colorless, morphologically slightly different derivative of **F**. **F **and **Fw **colonies are characterized by a typical massive rim, hence *rimmed*, in contrast to *rimless *(**R, W**) colonies. Colonies of the parental **R **strain and all daughter clones have a *finite growth*, their diameter being in rimmed clones about 15 mm, in rimless ones about 20 mm (after 10 days' growth). Colonies ripen into final color and pattern by about 7^th ^day upon planting, while still growing slowly, to reach their final diameter by day 15 (Figure 1a).

As the **F **morphotype plays a central role in this study, its development deserves a closer scrutiny. No matter how the colony was planted, in days 1-3 it grows as a central navel: a compact body on the agar plate only slowly propagating sideways. This phase is followed in days 3-5 by spreading of the flat interstitial circle. Microscopic observations revealed a margin of extracellular material containing small swarms of bacteria at the colony periphery at this stage (M. Schmoranz, AM and FC, unpublished observations), a phenomenon well established in *Serratia *sp. (e.g. [8,13]). In days 5-7 this lateral propagation comes to end and the peripheral rim is formed; the central navel grows red in this phase. In following days, the rim also turns red and the growth proceeds towards a halt. The flat interstitial ring remains colorless (Figure 1).

**Figure 2:**
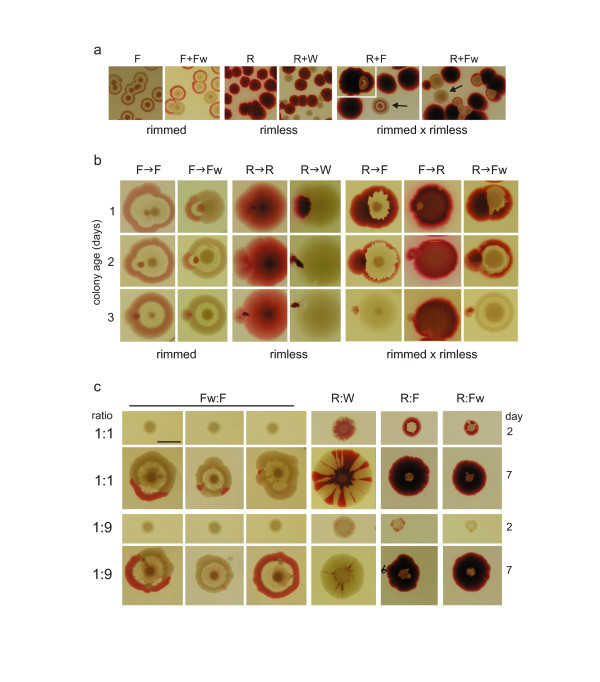
**Interaction of two bodies**. **a**. Colonies (at 7d, tens per dish) sown as single clones (**F, R**), or as mixtures of two clones (**F + Fw**, etc.). Note confluence in rimmed clones, as well as the **X **structures appearing in mixtures of rimmed-rimless clones (arrows). **b**. Planting (dotting) of a colony to the vicinity of a pre-existing colony (1, 2, or 3 days old, as indicated to the left); seen 3 days after dotting. Note strong pattern distortion in younger partner in all combinations; confluence of rimmed partners, in contrast to rimless ones; and encircling the rimmed partner by the rimless one. **c**. Plantings of mixed suspensions (cell ratio as indicated, bar = 1 cm). Two rimmed clones (**FFw) **give rise to highly variable structures resembling **F**-bodies, (three parallels from a single experiments shown). A mixture of rimless clones (**RW**) tends to maintain the identity of each clone. in combinations rimmed-rimless the rimmed partner becomes overgrown, albeit both cell types persist in the center.

Fully developed **F **colonies can be obtained only if bacteria are planted in densities 1-20 per 9-cm dish. At the density of tens per dish, the colonies grow much smaller; below a critical distance, they tend to fuse into a confluent colony with many centers bounded by a common rim (Figure 1b; see also Figure 2a). At densities of hundreds per dish, colonies remain very small and undifferentiated. Yet higher density of planting leads to a compact, undifferentiated body - a *macula *(Figure 1b). The scenario is similar for all four clones used in this study, except that rimless colonies (**R, W**) never fuse (Figure 2a). The development and behavior of standard colonies (as described above) were essentially independent on the way of planting (i.e., sowing, dropping, or dotting - see Methods), provided that the diameter of planting was ≤ 1 mm. Similar observations have been made previously for a different *Serratia *isolate [23].

### Close encounters of bacterial bodies

In dense plantings, clonal rimmed colonies tend to merge into confluent colonies (Figure 2a, left). In a mix of lines differing in color (**F, Fw**), the origin of each participating colony is revealed by the color of its rim. If colonies were dotted close (ca 1 mm) to a growing previously planted colony (24, 48, or 72 h old; Figure 2b, left), the resulting fused body resembled a confluent colony, with a common rim and separate centers. The effect was more profound with younger colonies.

If a similar protocol was followed with the rimless clones, colonies remained thoroughly delimited, whether in a single culture (**RR**), or in an **RW **mixture (Figure 2a, middle): no fusions were observed, and clearly distinguishable furrows developed between bodies in contact. Similarly, dots applied close to an older colony became inhibited in growth, but kept distinct from its growing older neighbor (Figure 2b, middle).

Upon close encounters between rimless and rimmed bodies (**RF **or **RFw**), the **R **colonies grow faster and influence rimmed colonies in four ways: (i) If planted early enough, **R **colonies can engulf an immature rimmed colony; its body, however, survives and cells can be recovered from such a mixed body (Figure 2b, right). (ii) If **F **colonies are allowed to grow in the vicinity of an **R **colony as independent bodies up to 3^rd ^day of cultivation (irrespective if they later grow to confluence or not), they develop to a *new colony phenotype *with a massive white rim with a thin colored ring at the inner side (a pattern dubbed **X **here and below; arrows in Figure 2a). Cells from **X **colonies restore the original **F **phenotype upon subsequent planting. (iii) A rimless (**R**) dot added to a rimmed colony (**F **or **Fw**) tends to proliferate around the older partner and block its further growth (Figure 2b, right); the younger the rimmed colony is, the more profound the effect. Similar results were obtained with **W **dots (not shown). Again, the rimmed colony remains compact (though overgrown) and contains live cells. (iv) The engulfment potential of the rimless colony is even more profound in a reverse arrangement, i.e. dotting of a rimmed colony to an older rimless partner (Figure 2b, right).

### Planting of mixed suspensions

Mixed suspensions of two rimmed clones (**F, Fw**) produced varying and unpredictable colony patterns (Figure 2c, left), suggesting an extreme sensitivity of such mixtures to initial conditions (e.g. minor inhomogeneities in the suspension). Samples taken from both center and periphery of such chimeras revealed the presence of cells belonging to both clones in the central zone, and sometimes also in the periphery (not shown). These results contrast with previous findings on a different strain [23]: in that case, however, both subclones tended to establish separated "areas of influence", essentially as referred below for **RW **mixtures.

If a colony was established from a mixture of two rimless clones **RW**, the center of the colony remained a mixture of both clones, sending radial monoclonal sectors as the colony grew (Figure 2c, middle), as if rimless clones were reluctant to cooperate towards a common end.

If a mixed suspension of rimmed (**F**) and rimless (**R**) suspension is dropped to initiate a colony, the cells of the rimmed clone remained confined to the central area, whereas the growing periphery is composed exclusively of **R **cells (Figure 2c, right), similar to the above-described engulfment of rimmed colonies by rimless ones. Again, the inhibited strain confined to the center remains viable and can be recovered upon re-planting. The behavior of **RFw, WF **and **WFw **colonies is analogous to the **RF **mixture (not shown).

### Effects of planting layout

**Figure 3:**
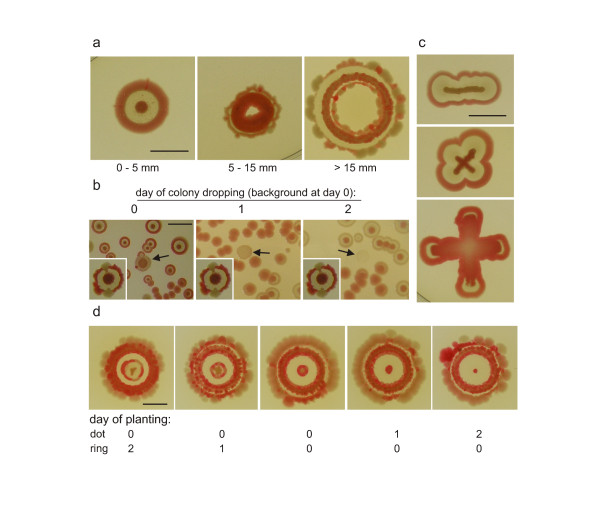
**F colonies developing from inocula of varying geometrical layout**. **a**. Colonies from ring inocula blotted using plastic matrices of varying diameter. **b**. Colony planting (1 μl, ca 10^5 ^cells) on the colony background of bacteria (0, 1, or 2 days old). Insets: controls. **c**. Simple cases of elongated plantings. **d**. Ring-colony encounters. Mutual influencing of a colony and a ring planted in different time intervals. All colonies are shown at day 7; bar = 1 cm.

The plasticity of the typical **F **body plan was investigated by streaking or blotting cell suspension in various geometrical settings. If the width of the plant in one direction does not exceed a critical diameter somewhat smaller than the adult **F **colony diameter, the body strives to maintain the features of the colony (i.e. colored center, interstitial zone, and rim), even if deformed to a large extent (Figure 3c). Blotting of ring bodies using circular plastic stamps was even more informative, with results depending on the diameter of such rings (Figure 3a; compare to Figure 1a). Smaller rings healed the central cavity and proceeded towards a normal (or almost normal) colony shape; with increasing diameter, up to the critical size, this colony phenotype was maintained, even if with a central hole in the middle. Above the critical diameter (15 mm), a ring-like colony acquired an additional inner rim - resembling linear colonies (streaks) as in Figure 3c, but curled.

We have also confirmed the previously described phenomenon of "ghost" colonies [23], originally documented on a different strain. Briefly, colonies planted at the background of multiple (hundreds) colonies became inhibited, or even "dissolved" on the background (Figure 3b). This is the case even in synchronous cultures if, at the beginning, the background is represented by at least about 100 colony-forming units. Such a background can keep at bay a plant as dense as 100 000 cells, preventing its development towards a colony. The effect is more profound when background colonies are older. With this information in mind, we return to ring colonies.

A colony was planted into the center of a ring colony of greater diameter, or a ring colony was blotted around a growing **F **colony. Both bodies represent a "background" to each other, depending on the succession of plating. Results in Figure 3d show that the synchronous planting of both structures leads to disruption of the structure of the central colony, but no change in the structure of the ring. Colonies planted on the background of older rings became inhibited. On the other hand, when the ring is planted around an older colony, it develops into a typical structure, only with more profound reddening of the inner rim - again confirming that a developing colony can perceive the presence and layout of its neighbors.

### Long-distance interactions between colonies and maculae

To examine the putative long-distance signals between bacterial bodies, colonies (**F**) were planted to the vicinity of maculae of two different *Serratia *clones (**F**, **R**) or an unrelated bacterial strain (*E. coli*). Maculae and colonies either shared the same agar plate, or were separated by a septum.

**Figure 4:**
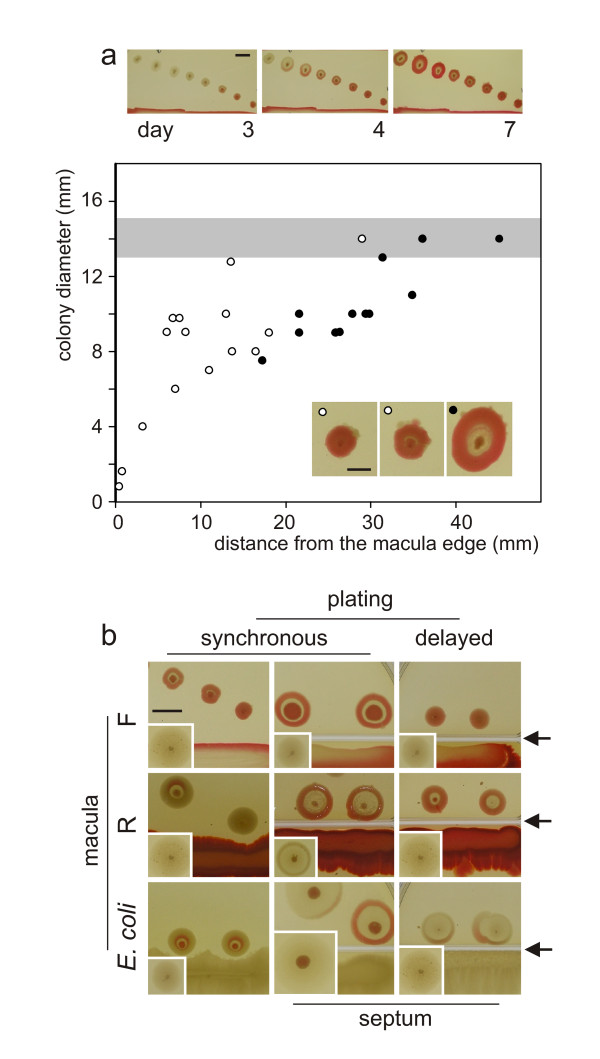
**F colony development in the presence of macula**. **a.F**-colonies planted simultaneously with an **F**-macula (12 cm dish). Top: overall geometry and time-course (macula edge at the bottom, bar = 1 cm). Below: colony pattern distribution at day 7; filled dots - standard **F **colonies; open dots - imperfect **F **pattern (see inset; bar = 5 mm); grey zone: interval of colony diameter in controls (no macula). Note the critical distance of ca 18 mm indicating the breakdown of typical **F **structure. **b.** Effect of maculae of different origin (as indicated) on the development of **F **colonies. Left column: synchronous planting, common space. Middle and right: macula separated from colonies by a septum (arrow), but sharing the gas phase. Middle: synchronous planting. Right: colonies planted to 3d macula. Insets: controls without maculae. Day 5 after colony inoculation, bar = 1 cm. In settings without a septum containing **R **or *E. coli *macula, note development of **X **phenotype.

When **F **colonies were planted in varying distances from an **F **macula (Figure 4a), the closer was the macula to a colony, the quicker the reddening of that colony. At the same time, the colony deviated from its typical structure to an extent inversely related to its distance from the macula. The graph in Figure 4a shows that the transition point between aberrant and standard patterns lies approximately 15 to 20 mm from the macula, corresponding roughly to the diameter of adult **F **colonies. This breakdown of the colony structure was not observed with the *Serratia *isolate characterized previously ([23]; data not shown). The **Fw **macula exhibited weaker effects than its **F **counterpart, and elicited the loss of structure only when older (not shown).

The **R **macula, as well as a macula of *E. coli*, induced, again, formation of the **X **phenotype in colonies of the **F **clone (Figure 4b, left; compare to Figure 2). No such **X**-like structures were observed when **R **colonies were planted in the vicinity of an *E. coli *macula (not shown).

### Communication across obstacles

**Figure 5:**
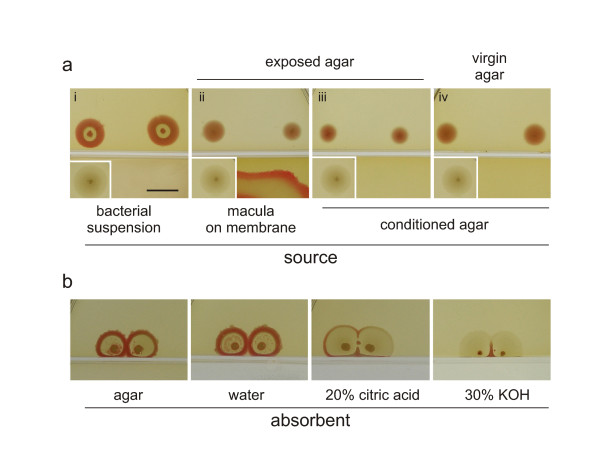
**Manipulating F colonies via the gas phase**. **a**. Cross-septum effects. Colonies are shown 4 days after planting into a compartment of a septum-divided Petri dish containing in its other compartment (i) bacterial (**F**) suspension; (ii) **F-**macula previously grown for 3days on a cellulose membrane; (iii) macula-conditioned agar obtained by growing a macula as in (ii), but removing it (with the membrane) immediately before colony planting; (iv) macula-conditioned agar obtained as in (iii), but colonies planted on a virgin agar (i.e. the agar medium in the colony compartment has been exchanged prior to colony plating). Insets: controls grown with uninoculated agar across the septum. **b**. Cross-septum effects (8 days after planting) of free agar, water, 20% citric acid, or 30% KOH (5 ml each). Bar = 1 cm.

If the macula and colonies have been grown on opposite sides of a septum dividing the dish, preventing diffusion in the semi-solid agar matrix but allowing gas exchange, the effect of macula was qualitatively similar to that on a shared plate, albeit the distance between the bodies appeared as if increased for simultaneously planted bodies (Figure 4b, middle). If, however, the macula across the septum was at least 3 days old at the time of colony inoculation, colony development was similar to controls sharing a continuous plate (Figure 4a, insert), suggesting that older bacterial bodies produce volatiles that may be absorbed by the agar medium. Maculae of a different strain (**R**) or species (*E. coli*) also affected development of **F **colonies across an obstacle; however, they never induced formation of the **X **structure across the septum, indicating that signals diffusing through the semi-solid substrate, distinct from those carried by the gas phase, are indispensable for the development of the **X **pattern. The effect across the septum is not bound to an organized *body *of the macula: bacterial suspension (**F**) kept across the septum exerted an effect comparable to that of a macula (Figure 5a; compare to Figure 4b).

### Nature of signals between bodies

In further experiments, we investigated the longevity of a putative macula-derived signal. A macula was grown for 3 days on a cellulose membrane laid on the agar on one side of a septum, then removed, leaving empty *macula-conditioned *agar. Immediately after macula removal, colonies were dotted into the neighboring compartment containing free *macula-exposed *agar (i.e. agar that was exposed - across the septum - to volatiles from the membrane-grown macula; Figure 5a). The results are indistinguishable from controls shown in Figure 4a, i.e. from the situation when the macula persisted in the neighboring compartment.

To test the obvious possibility that such free, but macula-exposed agar "took the smell" during macula growth, medium in the non-inoculated compartment was removed at the time of the macula removal, and replaced by "virgin" agar transferred from another, empty plate. As also shown in Figure 5a, the development of colonies was essentially the same as on macula-exposed agar. Thus, macula-conditioned agar can release sufficient amount of signal to influence the colony development on virgin agar. However, macula-exposed agar alone was unable to pass the effect further, to the virgin agar in the neighboring compartment (not shown).

The effect of conditioned agar suggests that the signals between bacterial bodies are chemical rather than physical (e.g., electric or electromagnetic pulses and/or vibrations such as sound). Since the effects is transmitted in the absence of living source bacteria, the most obvious candidate is some compound(s) soluble in the agar medium, readily evaporating (from the macula-occupied or conditioned agar), diffusing across the septum and becoming trapped in the free agar beyond. To exclude the possibility of transmission via surface of the septum, we rendered the septum hydrophobic by medical-grade vaseline (Herbacos-Biofarma). Since this did not affect the outcome of the experiment (not shown), we are left with the hypothesis of an airborne compound playing the role of the carrier of signal (or sign) for the recipient colony.

In a preliminary experiment, we tried to remove such putative compound(s) by placing possible absorbents into an adjacent compartment (Figure 5b): agar (control), water, 20% citric acid solution, or 30% KOH. As shown in Figure 5b, both citric acid and KOH appeared to be powerful inhibitors of colony development, while water or agar exhibited no effect.

### Modeling colony ontogeny

**Figure 6:**
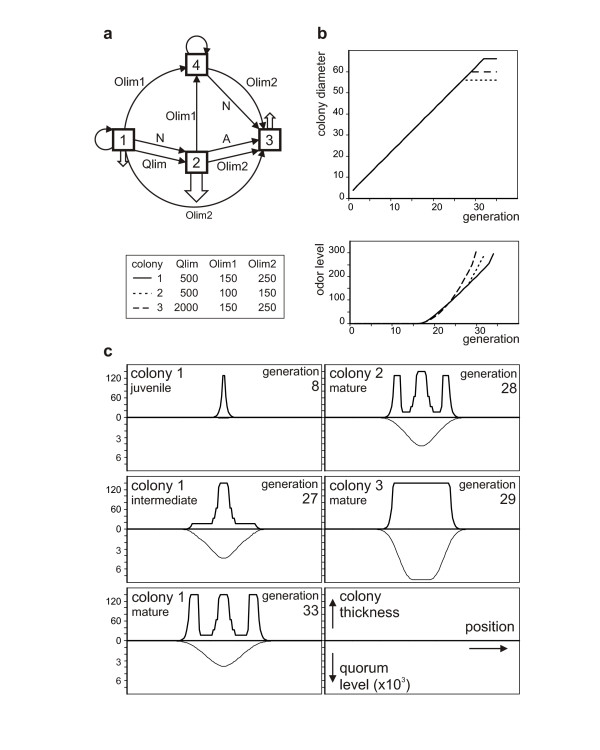
**The model**. **a**. Possible states and state transitions of bacterial cells,. All transitions allowed by the formal model are shown, regardless whether they take place during normal colony development; open arrows indicate production of quorum (downwards; arrow size is proportional to the intensity of production) and odor (upwards) signals. Each transition is labeled by the triggering factor (N - colony thickness, A - time spent in early stationary phase, Qlim - limiting quorum concentration, Olim1 and Olim2 - limiting odor level). **b, c**. Development of simulated rimmed and rimless colonies. Temporal development of colony size and odor level (**b**), and colony sections and quorum concentration profiles at selected points during colony development (**c**). All values are in relative/arbitrary units. Quorum and sensitivity parameters (quorum limit for inhibition Qlim, limiting odor concentration for growth reactivation Olim1 and limiting odor concentration for growth inhibition Olim2) for the simulations are shown in the figure. Other simulation parameters were: maximum colony thickness N = 140; quorum production factor P = 1; odor production factor O = 0.01; stationary to exponential quorum production ratio S = 10; quorum production window A = 5; normalized diffusion factor D = 0.495; diffusion approximated by G = 5 iterations.

We chose the process of development of the **F **colony pattern as a model case for establishing a causal scenario that might account for at least some of the processes leading to the development of intricately structured bacterial bodies. Our observations suggest involvement of volatile signals spreading through the gas phase and absorbed by the agar medium. At the same time, production of diffusible compounds spreading through the substrate by bacterial bodies is both well documented in the literature (see Discussion) and convincingly demonstrated in at least some of our experiments (note gradients of red pigment around **R **colonies in Figure 2a and 2b, as well as the development of **X **colonies). We thus proposed the following model, which includes both volatile (airborne) and diffusible (agar-borne) signals. It has been successfully implemented in a computer program simulating the temporal development of the **F **colony cross-section profile (Figure 6; Additional file 1; see also Methods).

In the course of the **F **colony development, a bacterial cell enters a succession of distinct states as follows (Figure 6a). In *State 1*, corresponding to freshly inoculated or "young" growing cells, the bacteria divide exponentially, resulting in a juvenile colony increasing in both its height and diameter. Cells in state 1 produce moderate amounts of a diffusible factor (further referred to as the "quorum") that spreads slowly through the substrate and inhibits their own growth if above a threshold concentration (Qlim in the model). When reaching Qlim, or as a result of nutrient limitation (approximated by a maximum colony thickness N in the model), cells stop dividing and enter *State 2*, corresponding to the early stationary phase and characterized by increased production of the quorum signal. At this stage, the developing colony consists of a core of non-growing cells in state 2, with a margin containing still-growing state 1 bacteria. Remarkably, over a range of parameters, thickness of the margin becomes soon limited by the quorum signal, while the colony keeps spreading laterally, resulting in a "hat-like" profile (not shown). Cells remain in state 2 for a limited time window (until reaching the "age" A), and then move on to *State 3 *- the mature stationary phase, where the production of the quorum signal ceases altogether but the bacteria start to emit another signaling compound - the volatile "odor" signal that is produced into the gas phase and readily absorbed into the agar across the whole dish (so that its concentration at any place reflects the total sum of production by all state 3 cells). Both state 1 and state 2 cells respond to a limiting concentration of the odor signal (Olim1) by entering *State 4*, or a refractory growing state, where the bacteria either keep dividing (if previously in state 1) or restore division (from state 2), but no longer produce any signaling compounds. They also do not respond to the quorum signal any more, while retaining sensitivity to the odor. Finally, upon reaching either the maximum colony thickness (N) or a second odor threshold (Olim2), state 4 cells cease growing and enter mature stationary phase (state 3), finishing thus colony development.

Computer simulations based on these assumptions yielded often colony profiles reminiscent of the observed behavior of **F **colonies (for an example see Figure 6b, c colonies 1 and 2). We cannot yet provide any rigorous estimate of the robustness of the **F**-like outcomes, as we have not systematically examined the space of model parameters; the reader is invited to do so using the provided program (Additional file 1). We obtained, however, "realistic" looking outcomes, though sometimes with distorted ratios of central, interstitial and peripheral colony zones, with a variety of parameters. We thus hope that the model might adequately describe a general aspect of the colony morphogenesis rather than an fortuitous outcome of a specific combination of parameters. Moreover, we were able to generate a "rimless" (**R**) phenotype solely by modifying the quorum and odor sensitivity limits while all the other parameters have been kept constant (Figure 6b, c colony 3).

### Simulation of specific features of rimmed colonies

**Figure 7:**
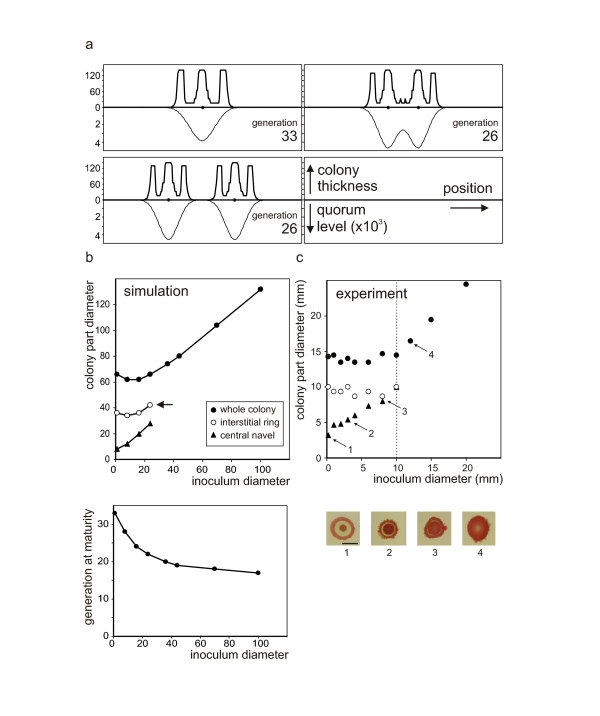
**Simulation of inoculum geometry effects**. **a**. Encounters of rimmed colonies. Profiles of mature colonies (including quorum levels) in the first generation after growth cessation. Inoculum position indicated by black dots. Colonies sharing the same substrate are smaller and reach maturity sooner than singletons, and develop a common rim if planted sufficiently close together. **b**. Effects of inoculum size in simulated plantings by dropping. Top - number of generations required to reach final colony size, bottom - diameter of distinct colony parts depending on initial inoculum size. Note that the simulation marked by the arrow resulted only in an imperfect, shallow rim, and simulations with larger inocula yielded maculae without a distinctive rim. Simulation parameters were as for colony 1 in Figure 6b, c. **c**. Experimentally observed dependence of colony proportions (at day 7) on area of planting. Increasing the planting area leads to the expansion of the red center at the expense of the interstitial circle. Above 10 mm of planting diameter (i.e. standard diameter of the circle; dashed line), the circle disappears totally, and the resulting body grows towards a macula.

While experimenting with varying layout of the initial inoculum (using parameters that generated rimmed colonies), we have observed three worthwhile additional phenomena (Figure 7a, b): (i) multiple inocula sharing the same dish developed into colonies of perfect shape but smaller size (compare Figure 1b); (ii) under some circumstances, colonies initiated close to each other "developed" a common rim (compare Figure 1b and Figure 2a); (iii) a simulation of dropping or dotting an extended inoculum yielded "rimmed colonies" from inocula smaller than the interstitial ring of a single cell-initiated colony but maculae for larger inocula. We have observed a similar phenomenon also in laboratory experiments aimed at estimating the area of planting that still allows cells in a slurry on the surface of semi-solid medium to coordinate their behavior.

Drops of dense suspension of the **F **strain were planted as smears of increasing diameter. As shown in Figure 7c, up to a critical diameter, roughly corresponding to the outer diameter of the interstitial circle of a normal **F **colony, the cells could still coordinate their actions towards a full-fledged colony, albeit not with a full success. If compared with the standard **F **pattern, the central navel always occupied the whole area of planting, leaving to the interstitial ring only the space remaining to the critical diameter. Should the diameter of planting reach (or exceed) this critical diameter, no room was left for the interstitial circle, and the body turned into a macula, as predicted by our formal model.

## Discussion

Highly structured bacterial bodies (mats, plaques, stromatolites, colonies, etc., containing astronomical amounts of cells belonging to hundreds of species) apparently represent the "default" way of living of most bacteria [25-34]. How do such bodies come into existence? Are they *ad hoc *contraptions, molded solely, or predominantly, by the external environment? A result from an ecological succession, a game played by well-trained players? Or, finally, may an analogy of ontogeny be assumed [23], similar to ontogeny in, e.g. mycobacteria, streptomycetes, slime molds, yeasts, or even plants or animals?

Our experiments with a single clone or a pair of clones, each giving well-developed colonies with finite growth, may provide initial insight into the processes of bacterial body formation. Apparently, there exists an elaborated network of communicative signals mutually affecting bacterial bodies, so the first hypothesis can be safely dismissed. As to the "ecology" of cells: a colony, whose diversity resembles rather a carefully managed cornfield than a natural landscape, may not seem to be a good model at the first sight. Yet, our two interacting clones remind of theoretical models like "hawks-doves" or "prisoners dilemma" (or even interaction of a monoculture with weeds or pathogens). The third possibility - regular patterns of colony ontogeny - allows even genuine convergent development entering the game.

Our observations suggest that development of bacterial bodies in *Serratia *sp. includes both events taking place within a body, and transmission of signals between distinct bodies. Signals act at a distance, i.e. they do not require physical contact (as reported, e.g., in [16,35,36]). Experiments with conditioned agar show that signals do not require simultaneous presence of living entities; hence, actively emitted light, sound, electrical or even chemical pulses of whatever nature can be excluded as carriers of the signal. We are left with a compound or a cocktail of compounds, emitted *by *living entities into their environment, persisting there for some time, and being actively interpreted by recipients that happen to be present in their range. While our observations do not provide any hints yet as to the chemical identity of these signals, they at least point towards some of their properties. Experiments with signaling across the septum suggest that the signal from the macula spreads via the gas phase. For a different bacterial system, indole could be the carrier of a volatile signal ([37]; however, this conclusion was later questioned [38]). Ammonia appeared to be the signal carrier in yeast colonies [39]. As a first step towards characterizing our signal, we demonstrated that it is readily cleared away by non-volatile acid or alkali traps.

We propose a simple model capable of simulating some aspects of our experimentally characterized examples of bacterial body morphogenesis (the **F **and **R **colonies). This model involves *two factors *carrying information for both morphogenesis and mutual influencing of neighbors, generated in bodies at certain developmental stages, and diffusible to the environment. One of the signals travels (slowly) through the substrate, the other is transmitted via the gas phase. These bearers of the signals (or even a sign) are perceived by all cells, allowing their orientation and behavior in the developing colony; timing may be the second critical factor at play. While several theoretical models of microbial colony morphogenesis have been published, they mostly focus on such aspects as kinetics of colony expansion controlled by nutrient diffusion through the colony and surrounding medium [40-43], intra-colony spatial organization of cells [44,45] or fine patterning of the colony margin based on interplay of nutrient and signal diffusion and, in some cases, also swarming behavior of the bacteria [46-48]. To our knowledge, none of the published models accessible to us predicts or explains formation of "fountain" colonies of finite size (although some predict repetitive ring patterns - e.g. [49-51]), and none includes an interplay of diffusible (substrate-borne) and volatile (air-borne/substrate-absorbed) signals, albeit chemotaxis or quorum sensing has been incorporated in some simulations (e.g. [44,45,50]).

So far, our model does not account for modifications of the colony's "body plan" upon interaction with different clones (or even species), where additional signals diffusible in agar (or modulation of the response(s) to one signal by the other), may contribute (e.g. our **X **pattern, or mutual inhibition occurring upon encounter of two rimless colonies; the later has been explained by others [43] as a possible consequence of bacteria interpreting local nutrient concentration as a signal inducing growth rate changes). Notably, our model includes, as one of the central parameters, some kind of cellular memory - bacteria that have recently ceased dividing behave differently from their sisters that have spent a longer time in the stationary phase.

Let us suppose that in closely related bacterial clones used in our study the basic morphogenetic signals are the same, i.e. particular clones differ in the signal interpretation. Remarkably, some combinations of quorum and odor sensitivity parameters in our model produce rimless bodies while other parameters are kept the same as for rimmed ones (Figure 6).

Changes in the rate of lateral spreading during colony development have been observed or predicted especially for microbes exhibiting extensive swarming; however, we have not incorporated this phenomenon into our model since both our observations (Figure 1) and data reported by others [47] document a more-less constant rate of lateral growth of *Serratia *colonies under conditions leading to the development of compact colonies (as in our study).

The present model does not yet allow simulations involving more than one "clone" (defined by a specific set of parameters). Nevertheless, the experimentally observed "aggressive" phenotype of rimless bodies upon encounter with rimmed ones is consistent with the model assuming that the rimless clone is less sensitive to the (inhibitory) diffusible quorum signal spreading through the substrate. A "rimless" phenotype has been previously observed also in a *S. marcescens *strain capable of forming "fountain" colonies on standard media, when this strain was grown in the absence of glucose [23]; the same happened also in our **F **clone on glucose-free media (data not shown). It is tempting to speculate that glucose (or another effective energy source) may be required to develop full sensitivity to the diffusible quorum signal. Alternatively, production of such a signal may be diminished on poor media (since what matters in our model are not absolute values of quorum signal production but the ratio between production per bacterium and the sensitivity threshold value).

The difference in "worldviews" between rimmed and rimless clones is best demonstrated when mixed suspensions or colonies planted close together are forced towards establishing a new body. The rimless partners segregate in radial clonal sectors from a mixture, and keep separated upon close encounter. On the other hand, two rimmed clones are much closer to each other in interpreting their morphospace than two rimless clones, as they can build a common rim when planted as a mixed suspension or upon close encounters.

We have experimentally defined several additional qualitative prerequisites for establishing and maintaining the typical "body plan" of bacterial colonies; some of them can be evaluated in the light of our model.

The presence of a bacterial body in the neighborhood of a developing colony of **F **clone results in its quicker ripening, i.e. reddening. Very close encounters lead to disruption of both its growth and pattering: most profound is the effect on colonies planted close to older bodies, or inside ring-bodies. In case of two rimmed partners, the older the neighbor was, the more profound the growth inhibition of the younger colony, which, nevertheless, remained recognizable even when overgrown by the older partner.

Development of geometrically constrained bodies (such as those originated by ring-shaped, elongated or cruciform inocula) can be interpreted as a conflict of two ways of recognizing the "body" across the hole: as a part of "self" (resulting in a symmetric colony, or a colony with a hole, for small rings), or as a neighbor. In ring plantings up to a certain diameter, cells in the inner diameter of the ring are sufficient to produce a "virtual navel" controlling the development of the body. In large rings, the "non-self" tendency prevails: such bodies take the inner empty space for outer space outside of their morphogenetic field. New colonies planted into such an area are treated as foreign, and their pattern resembles those planted in the vicinity of other type of bodies.

While our model does not currently allow simulating development of multiple inocula differing in genotype (i.e. parameters), size, shape or time of planting, we could at least reproduce the faster ripening and smaller size of two colonies sharing a confined space, compared to a solitary colony.

We have also confirmed our previous results [23] showing that the growth of colonies is strongly inhibited, even abolished, if the surrounding area is evenly occupied by "background" bacterial bodies - even if their total population (biomass) is much smaller than the colony inoculum. Hence, bacteria in the background emit a signal that efficiently disturbs the organizing potential of the multicellular plant, while keeping the background colonies in an underdeveloped - "dormant" - state. Experiments with effects of colonies grown in the presence of maculae (sharing the substrate or separated by a septum), macula-exposed and "virgin" agar suggest that this effect could be identical with the gas-borne signal of our model.

Finally, we have shown that the planting area necessary for the cell population to maintain the "feeling" of belonging to a single body, roughly corresponds to the outer diameter of a mature interstitial circle (Figure 7c). Exceeding this critical diameter leads to the loss of structure and breakdown to a macula; however, even in such a case the body is self-inhibited as to lateral spreading. This may perhaps be understood as the last remnants of its "feeling of integrity"; the results of our computer simulations suggests that even this seemingly complex effect may be produced by the interplay of mere two signals.

## Conclusions

Some isolates of *Serratia *sp. produce colonies exhibiting finite growth and clone-specific appearance, which is easily evaluated thanks to their conspicuous coloration. The shape and patterning of developing colonies and other multicellular bodies is easily malleable by experimental conditions. The appearance of a developing colony results from (i) its internal morphogenetic potential and (ii) the character of neighbor bodies and their overall distribution on the dish.

A simple formal model is proposed, based on two morphogenetic signals generated by the bodies, one of them spreading through the substrate and the other through the gas phase. The model can simulate some of our experimental results, namely:

1. The development of colonies exhibiting finite growth and both rimmed and rimless patterns, the difference between the former and the latter being in the intensity of signal production and/or sensitivity towards the signal(s).

2. Dependence of colony size upon the number of colonies sharing common morphospace, and development of confluent colonies from closely planted inocula of a rimmed strain.

3. The phenomenon of "critical planting area" which must not be exceeded should a colony develop a typical rimmed pattern.

Our observations are thus consistent with bacterial colonies behaving, in some aspects, as true multicellular bodies whose patterning is controlled by positional information; the nature of the relevant signals remains to be established.

## Methods

### Strains, media and culture conditions

The strain *Serratia rubidaea *here labeled **R **(rimless "wild type" phenotype for the purpose of this study), as well as *E. coli *strain 281, were obtained from the collection of the Department of Genetics and Microbiology, Faculty of Sciences, Charles University. The **R **strain, originally described as *S. marcescens*, has been determined as *S. rubidaea *on the basis of metabolical markers and gyrB gene sequencing (A. Nemec, National Health Institute, Prague, personal communication). The remaining three *Serratia *sp. clones are morphologically distinct derivatives of **R **selected after prolonged serial cultivation in liquid media with subsequent plating; the **F **and **W **clones have been confirmed to be derived from the **R **clone by comparison of SpeI RFLP patterns obtained using pulsed-field gel electrophoresis (A. Nemec and M. Schmoranz, personal communication). Details of the strain genealogy and characterization will be reported in a future study focused on variability of *Serratia sp*. colony morphology (M. Schmoranz, Z. Neubauer, AB and AM, in preparation).

Bacteria have been grown under previously described standard conditions [23] on Nutrient Agar No2 (Imuna Pharm a.s., Order No T 382100001020) supplemented with 0.5% glucose, or on a medium obtained by solidifying Nutrient broth No2 (Imuna Pharm a.s., Order No V 382100000098) by addition of 1,5% agar, supplemented with 0,5% glucose, with the same results. The standard colony patterns have been also reproduced on standard LB medium with 0.5% glucose (not shown). Bacterial stocks have been maintained at -80°C as described previously [23].

New colonies were initiated (1) as clones from single cells, by classical *sowing *of bacterial suspension (in phosphate buffer); (2) by *dropping *such suspension on a defined place; (3) by *dotting*: from material taken by a sterile needle from an older body; (4) by *streaking *a mass of bacteria from an older colony using a sterile bacteriological loop; (5) by *blotting *from a continuous carpet of bacteria using plastic matrices of required shape (made of disposable plastic tubes or pipette tips). To obtain conditioned agar, the agar plate was covered by cellulose membrane (Blanka, CSN 646811, Chemosvit), and macula was sown (by dropping) on top of the membrane. After 3 days, cellulose membrane with bacterial mass was removed. Signaling across compartments was studied in septum-divided Petri dishes providing isolated agar compartments, but sharing the gas phase (Gama Group a.s., order No 400901).

### Documentation

Plates were photographed *in situ *using an Olympus digital camera under ambient or penetrating light (Fomei, LP-400 light panel, cold cathode light) or under magnification using a binocular magnifier. Figures shown were selected from an extensive collection of primary photos from several repetitions of each experiment. Photoshop software was used to assemble the plates but no image doctoring was performed except automatic adjustment of brightness and contrast in some cases.

### Mathematical modeling

The model (see Additional file 1) has been developed and modeling performed in the freely available Python 2.6.4 environment [52] on a Windows-based PC. The model is designed as a one-dimensional continuous cellular automaton, where the row of "cells" represents a projection of the developing colony cross-section onto a level parallel with the substrate surface. Each "cell" is characterized by discrete values of (i) bacterial layer thickness (number of bacteria), (ii) state of the bacteria (depending on local conditions and in some cases also recent history; see Results), and (iii) in case of recently stationary bacteria also their "age", i.e. time elapsed since growth cessation. Two continuous variables represent (iv) concentration of the diffusible "quorum" signal in the substrate and (v) amount of the volatile "odor" signal that is by definition equal across all cells. The model develops in a series of generations, each consisting of four steps: (1) evaluation of the state of bacteria in each cell according to their age (if defined) and concentration of quorum and odor signals; (2) division of bacteria in each cell according to their state, followed by migration of one daughter bacterium into the neighboring cell if this cell is empty and if no limitation by diffusible factors occurs; (3) production of quorum and odor signals by bacteria in each cell; (4) diffusion of the quorum signal, itself approximated by a nested multi-step process where each step represents migration of a fixed fraction of the difference in quorum signal concentration down the concentration gradient between each two neighboring cells. Raw data produced by the model have been evaluated and graphically represented using MS Excel.

## Authors' contributions

JC and IP contributed equally to the designing and performing the experiments and interpreting their results; FC developed the formal model and participated in writing the paper; AB participated in experiments and data interpretation and provided basic technical support; AM participated in study design and data interpretation and drafted the paper. All authors have read and approved the final manuscript.

## Supplementary Material

Additional file 1 **Formal model of colony patterning (colony1.py)**. A Python program file that can be run in the Python 2.6.4 environment (freely available at http://www.python.org). The program is annotated in a human-readable form, accessible using any text editor. Click here for file
